# Comparison of Phenotypic Characteristics and Antimicrobial Resistance Patterns of Clinical *Escherichia coli* Collected From Two Unrelated Geographical Areas

**DOI:** 10.5539/gjhs.v6n6p126

**Published:** 2014-07-15

**Authors:** Mutasim E. Ibrahim, Naser E. Bilal, Mohamed E. Hamid

**Affiliations:** 1 Department of Medical Microbiology, Faculty of Medical Laboratory Sciences, University of Khartoum, Sudan; 2 Department of Clinical Microbiology and Parasitology, College of Medicine, King Khalid University, Abha, KSA

**Keywords:** antimicrobials resistance patterns, comparison, *Escherichia coli*, unrelated geographical areas, Saudi Arabia, Sudan

## Abstract

**Background:**

Antimicrobial resistance among pathogenic *Escherichia coli* is an increasing problem especially in developing countries.

**Aims:**

To compare between resistance patterns of *E. coli* collected from two unrelated geographical areas.

**Methods:**

A descriptive comparative study was conducted between May 2010 and August 2011. *E. coli* (n= 402) collected from hospitals in Khartoum state, Sudan and in Aseer region, Saudi Arabia were studied. Identification and antimicrobial susceptibility testing of isolates were performed following standard methods. Multi-drug resistance (MDR) was defined as non-susceptibility to ≥ three antimicrobials.

**Results:**

Of the 402 *E. coli* isolates studied, MDR patterns were significantly higher among isolates from Sudan than Saudi Arabia [92.2% (214/232) vs. 70.6% (120/170)] (p = 0.000). The resistance rates of *E. coli* isolates were recorded as follows (Sudan and Saudi Arabia): High to moderate resistance to amoxicillin (97.7% and 94.2%), trimethoprim-sulfamethoxazole (88.3% and 82.5%), tetracycline (77.1% and 74.2%), amoxicillin- clavulanic acid (51.4% and 70%), ceftriaxone (64% and 52.4%) and ciprofloxacin (58.4% and 40%). Low resistance was to ceftazidime (35% and 20%), gentamicin (35% and 17.5%) and nitrofurantoin (22.4% and 11.7%). Resistance to amikacin was uncommon (1.9% and 5%). Significant differences (p < 0.05) in resistance rates of isolates between both countries in term to patient’s gender and age. The most frequent MDR phenotypes among isolates were to 7(15.9%) in Khartoum state and to 3(20.8%) in Aseer region.

**Conclusions:**

Variation and emerging of antimicrobial resistance among pathogenic *E. coli* isolates was observed in both regions. Continuous monitoring of resistance profiles, locally and international surveillance programs are required.

## 1. Introduction

*Escherichia coli* is a clinically significant bacterium because they are the most common species recovered in the clinical laboratories and has been incriminated in human infectious diseases (Koneman et al. 2006). During the last few decades, *E. coli* have evolved toward antimicrobial resistance (Nys et al., 2004; Peralta et al., 2007). The worldwide spread of *E. coli* strains with different multi-drug resistance (MDR) phenotypes make wide ranges of antimicrobials usefulness to treat most infections caused by this bacterium (Bilal et al., 2001; Saenz et al., 2004; Al-Tawfiq, 2006; Aminizadeh & Kashi, 2011). Antimicrobial resistance has been reported in developed countries (Sahm et al., 2001; Karlowsky et al., 2004; Oteo et al., 2005). However, resistance to microbial agents is growing very fast in developing countries such as in Africa (Okeke et al., 2007), Asia (Hsueh et al., 2008; Uma et al., 2009) and South America (Bartoloni et al., 2006). In Khartoum, capital of the Sudan, antimicrobial resistance patterns among clinical isolates were recorded as a major health problem (Hamdan et al., 2011; Ibrahim et al., 2012). In Aseer region, south west of Saudi Arabia, previous studies undertaken to determine the prevalence of various bacterial pathogens causing infections and their *in vitro* antimicrobial susceptibility profile (Bilal et al., 2001; Hamid et al., 2011). In a year 2001, a study was conducted in Abha Maternity Hospital, a teaching hospital in Aseer, south-west region of Saudi Arabia, about 30 different resistance patterns were shown by either the hospital or community isolates (Bilal et al., 2001). Increasing of international travelling from country to another have potential role in the spread and dissemination of resistant bacteria (Blomberg et al., 2004; Nys et al., 2004). However, comparative study of antimicrobial resistance of bacterial pathogens between countries and different geographical regions may be helpful in the evaluation of resistance problem (Blomberg et al., 2004). This study aimed to compare between antimicrobial resistance patterns of clinical *E. coli* isolates from patients at hospitals in Khartoum state, Sudan to those collected from hospitals in Aseer region, Saudi Arabia.

## 2. Methods

### 2.1 Study Design and Settings

This was a descriptive comparative study conducted during the period between May 2010 and August 2011. A total of 402 *E. coli* were isolated from various clinical specimens of patients of all age groups at six hospitals in Khartoum State, Sudan and two hospitals in Aseer region, southwest of Saudi Arabia. In Sudan, full details of *E. coli* isolates and participating hospitals have been described in our previous study (Ibrahim et al., 2012). While in Saudi Arabia, *E. coli* were collected from two hospitals including Aseer Central Hospital and Abha Maternity Hospital. The related hospitals in both countries were referral and educational hospitals, including different specialties and therefore serving various patient groups and covering the most population of different areas.

The study was laboratory based study and did not involve any intervention concerning the patients directly. All databases which included, specimen source and patient sex, age and setting were carefully recorded from laboratory request form. Therefore, ethical approval form did not obtained as per study guidelines.

### 2.2 Bacterial Isolates

The microbiology laboratory of each hospital undergoes the routine processing of the various clinical specimens of patients. Isolation and identification of pathogenic *E. coli* followed standard conventional procedures (Cheesbrough, 2000; Farmer, 2003).

### 2.3 Antimicrobial Susceptibility Testing

Antimicrobial susceptibility testing of *E. coli* isolates were performed by the Kirby-Bauer disk diffusion assay on Mueller-Hinton agar medium (Oxoid, Basingstoke, England) as recommended by Clinical Laboratory Standard Institute (CLSI, 2011), against 15 antimicrobial agents from different categories including: amikacin (30 μg), amoxicillin (10 μg), amoxicillin-clavulanic acid (30 μg), ceftazidime (30 μg), ceftriaxone (30 μg) cefuroxime (30 μg), chloramphenicol (30 μg), ciprofloxacin (5 μg), gentamicin (10 μg), nalidixic acid (30 μg), nitrofurantoin (50 μg), ofloxacin (5 μg), tetracycline (30 μg), tobramicin (10 μg) and trimethoprim- sulfamethoxazole (25 μg) (Oxoid, Basingstoke, England). *E. coli* isolate was considered non-susceptible to an antimicrobial agent when it tested resistant, intermediate or non-susceptible when using clinical breakpoints as interpretive criteria, provided by the CLSI, (2011). MDR patterns of *E. coli* isolates were defined as non-susceptibility to at least one agent in three or more antimicrobial categories (Magiorakos et al., 2012).

### 2.4 Statistical Analysis

Collected data were analyzed using Statistical Package for Social Sciences (SPSS; Version 10) software. Comparisons of antimicrobial resistance rate of isolates from both countries were done in term of setting, sex and age. The proportions were compared using the Chi-square test. A p-value of less than 0.05 was considered as statistically significant.

## 3. Results

### 3.1 Characterizations of E. coli Isolates

A total of 402 *E. coli* isolates (232 from Khartoum state, Sudan and 170 from Aseer region, Saudi Arabia) were examined for antimicrobial susceptibility testing using double-disk diffusion method against 15 antimicrobial agents from different categories. Out of the 402 *E. coli* isolates tested for their antimicrobial susceptibility, 334 isolates were characterized as MDR strains from the two geographical regions. The majority of MDR *E. coli* were recovered from specimens of urine (n = 220) followed by wounds (n = 59) and vaginal swabs (n = 20) with low isolation rates from other specimens (Table 1).

**Table 1:** Distribution of MDR *E. coli* (n = 334) collected from Khartoum state, Sudan and Aseer region, Saudi Arabia in relation to specimen types

Specimen type	Khartoum state, (Sudan) (n=214)	Aseer region (Saudi Arabia) (n=120)
	No. of MDR *E. coli* (%)	No. of MDR *E. coli* (%)
Urine (n =220)	135 (63.1)	85 (70.8)
Wound pus (n =59)	51 (23.8)	8 (6.7)
Vaginal swab (n =20)	7 (3.3)	13 (10.8)
Blood culture (n = 7)	5 (2.3)	2 (1.7)
Semen Fluid (n = 3)	3 (1.4)	0.0 (0.0)
Stool (n =11)	2 (0.9)	9 (7.5)
Ear swab (n =11)	8 (3.7)	3 (2.5)
Other body fluids (n =3)	3 (1.4)	0.0 (0.0)

Out of 232 *E. coli* strains collected from patients in Khartoum state, Sudan, 214 (92.2%) were found to be MDR *E. coli*. These 214 MDR *E. coli* isolates were obtained from all age groups: 125 (58.4%) were from females and 89 (41.6%) were from males. Of these 214 MDR isolates, 168 (78.5%) were obtained from adult patients, whereas 46 (21.5%) from children.

Of the 170 *E. coli* strains collected from hospitals in Aseer region, Saudi Arabia, MDR *E. coli* represented 120 (70.6%) of total isolates. These 120 isolates were obtained from females (n = 77) and males (n = 43). Eighty seven (72.5%) of the 120 isolates were from adults, whereas 33 (27.5%) were from children patients.

### 3.2 Frequency of MDR Patterns of E. coli

The occurrence of MDR patterns was found to be significantly higher among *E. coli* sourced from Khartoum state, Sudan than that from Aseer region, Saudi Arabia isolates [92.2% (214/232) vs. 70.6% (120/170)] (p = 0.000).

Table 2 summarized the resistance patterns of *E. coli* recovered from clinical specimens of patients in Kartoum state, Sudan and Aseer region, Saudi Arabia. The resistance rates among the strains from both countries were recorded as follows (Sudan and Saudi Arabia): High to moderate resistance rates of isolates were observed in amoxicillin (97.7% and 94.2%), cefuroxime (92.5% and 35.8%), trimethoprim-sulfamethoxazole (88.3% and 82.5%), tetracycline (77.1% and 74.2%), nalidixic acid (72% and 57.5%) ceftriaxone (64%). ciprofloxacin (58.4% and 40%), ofloxacin (55.1% and 38.3%), amoxicillin-clavulanate (51.4% and 70%). Low resistance rates of isolates were observed to ceftazidime (35% and 20%), gentamicin (35% and 17.5%), nitrofurantoin (22.4% and 11.7%), chloramphenicol (18.2% and 18.3%), and tobramicin (18.2% and 29.2%). Resistance to amikacin was uncommon (1.9% and 5%).

**Table 2:** Antimicrobial resistance patterns of *E. coli* strains collected from patients in Khartoum state, Sudan and Aseer region, Saudi Arabia

Antimicrobial agent	Khartoum state (n = 214)	Aseer region (n = 120)	P. value
	% Resistance	% Resistance	
Amikacin	1.9	5.0	0.108
Amoxicillin	97.7	96.7	0.591
Amoxicillin-clavulanic acid	51.4	70	0.001
Ceftazidime	35	20	0.004
Ceftriaxone	64	31.7	< 0.001
Cefuroxime	92.5	35.8	< 0.001
Chloramphenicol	18.2	18.3	0.980
Ciprofloxacin	58.4	40	0.001
Gentamicin	35	17.5	0.001
Nalidixic acid	72	57.5	0.007
Nitrofurantoin	22.4	11.7	0.015
Ofloxacin	55.1	38.3	0.003
Tetracycline	77.1	74.2	0.548
Tobramicin	18.2	29.2	0.021
Trimethoprim-sulfamethoxazole	88.3	82.5	0.140

As shown in Table 2, there were significant differences (p < 0.05) of antimicrobial resistance rates of *E. coli* isolates collected from Khartoum state, when compared to those from Aseer region. Strains from Khartoum state were more resistant to ceftazidime, ceftriaxone, cefuroxime, ciprofloxacin, gentamicin, nalidixic acid, nitrofurantoin and ofloxacin, whereas isolates fromAseer region were more resistant to amoxicillin-clavulanic acid and tobramicin. In general, Sudan sourced strains were more resistant to antimicrobial agents than those from Saudi Arabia.

### 3.3 Comparison of Antimicrobial Resistance in Relation to Patient’s Gender

**Figure 1:**
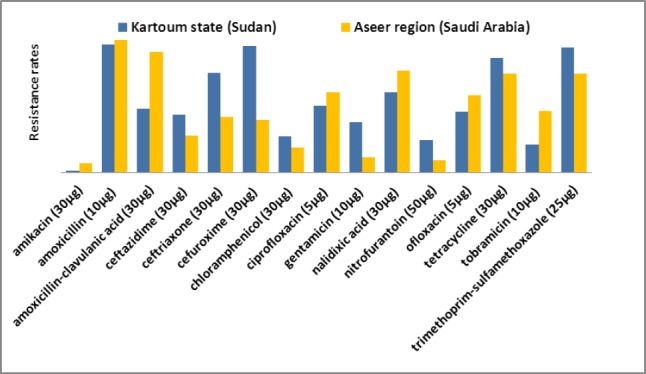
Comparison of resistance pattern of *E. coli* isolated from male patients in Khartoum state to those from Aseer region

Figure 1 shows the rates of resistance of *E. coli* strains recovered from male patients in Khartoum state, Sudan and Aseer region, Saudi Arabia. Higher percentage of resistance was reported among isolates from males in Khartoum state than those from Aseer region for ceftriaxone (p < 0.001), cefuroxime (p < 0.001), gentamicin (p = 0.002), nitrofurantoin (p = 0.037), trimethoprim-sulfamethoxazole (p = 0.001). In contrast, higher resistance rates among isolates from Aseer region were observed for amoxicillin-clavulanic acid (p < 0.001) and tobramicin (p = 0.003).

**Figure 2:**
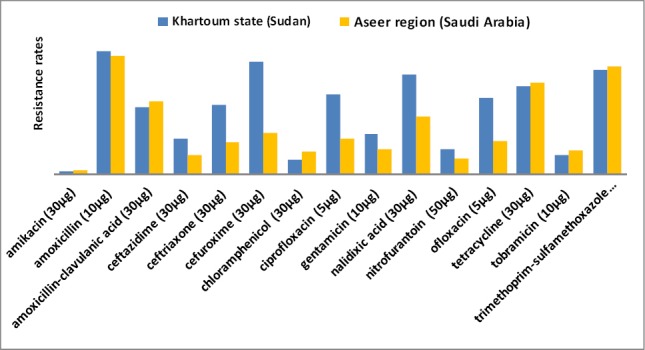
Comparison of resistance pattern of *E. coli* isolated from female patients in Khartoum state to those from Aseer region

When comparing the resistance rates of *E. coli* isolates recovered from female patients in Khartoum state to those fromAseer region (Figure 2), the incidence of cephalosporins and quinolones resistance were higher among females from Khartoum state than those from females in Aseer region, whereas no statistical differences were observed in the remaining tested antimicrobial agents.

### 3.4 Characterization of MDR Phenotypes of E. coli Isolates

**Figure 3:**
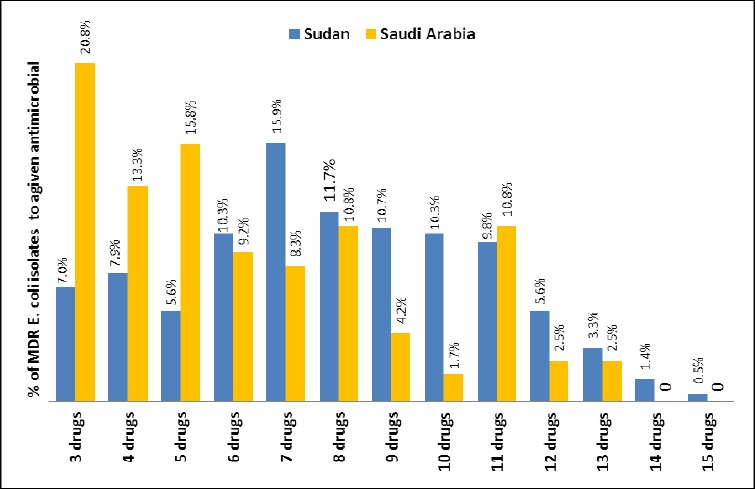
The percentage of *E. coli* isolates recovered from hospitals in Khartoum state, Sudan and Aseer region, Saudi Arabia showing different MDR phenotypes to a given number (3 to 15) of antimicrobial drugs

Figure 3 shows the resistant phenotypes of *E. coli* isolates collected from Khartoum state, Sudan and Aseer region, Saudi Arabia according to the given number (n = 3 to 15) of antimicrobial drugs. Among the isolates collected from Khartoum state, Sudan the most prevalent MDR phenotypes were to 7 (15.9%), followed by 8 (11.7%) of tested antimicrobial agents. While among the strains originated from Saudi Arabia, the most frequent MDR patterns were to 3 (20.8%) followed by 5 (13.3%) and 4 (15.8%) of antimicrobial agents.

The most frequent resistance profiles among Sudan strains were: AML-AMC-CXM-CRO-CAZ- CIP-OFX-NA-SXT-TE (8 strains) followed by AML-CXM-CRO-CAZ-CIP-OFX-NA-SXT-TE and AML-CXM-SXT-TE (7 strains), AML-CXM-CRO-CAZ-CIP-OFX-NA-SXT-TE-GN-TOB and AML-CXM- CRO-CAZ-F-SXT-C (6 strains each). Among Saudi Arabia strains, the predominant resistance profiles were: AML-SXT-TE (12 strains), AML-AMC-CXM-CRO-CAZ-CIP-OFX-NA-SXT-TE-TOB (10 strains), AML-AMC -SXT-TE (8 strains) and AML-AMC-NA-SXT-TE (6 strains).

## 4. Discussion

This study was focused on the antimicrobial resistance patterns of *E. coli* isolates recovered from two different unrelated geographical areas of Khartoum state in Sudan and Aseer region in Saudi Arabia. Antimicrobial resistance rates vary among regions and countries, with increasing rates in the many parts of the world (Blomberg et al., 2004; Bartoloni et al., 2006; Okeke et al., 2007; Hsueh et al., 2008; Uma et al., 2009; Ibrahim et al., 2012).In certain countries, the spread of resistance pathogens is mainly due to inappropriate use of antibiotics (Nys et al., 2004; Al-Tawfiq, 2006; Aminizadeh & Kashi, 2011; Okeke et al., 2007).Therefore, the availability of antibiotics without prescriptions, insufficient education of the medical profession about drugs, non-availability of guidelines for therapy and shortage in quality control measures are contributing in development and emerging of antimicrobial resistance (Hsueh et al., 2005; Awad et al., 2006; Awad & Eltayeb, 2007). In the present study, the MDR patterns of *E. coli* isolates from the Khartoum state hospitals, Sudan was found to be increasing (92.2%) when comparing to a survey done in Sudan in the year 2000 in which MDR were recorded as 58% (Ahmed et al., 2000). In a recent study in the Sudan, *E. coli* isolates from urinary tract infections with its multi resistance towards antimicrobials has been documented (Hamdan et al., 2011).Whilst MDR rates of *E. coli* in Aseer region, Saudi Arabia were recorded as 70.6%. This percentage was still of higher levels compared to previous study carried out in the same regions by Bilal et al. (2001). These authors reported MDR rates among clinical *Enterobacteriaceae* isolates as more than 74 % in the year 2000. Moreover, our findings were much higher than 39% found as MDR *E. coli* in another region of Saudi Arabia (Al-Tawfiq, 2006). These findings indicate that antimicrobial resistance pattern among clinical isolates is emerging in both geographical areas and could complicate the treatment of infections lead to serious health problem. The high rates of MDR patterns among the isolates in this study might be due to misuse and unnecessary prescription of antimicrobial drugs in both countries. In this study, we found that urine is a principle source of MDR isolates from both regions. In this study, we found that urine is a principle source of MDR isolates from both studied regions. As described by others (Sahuquillo et al., 2011), the existence of marked differences in the susceptibility to several antimicrobial agents depending on the site of the isolates. On the other hand, resistance patterns of isolates from abscesses, bloodstream, exudate, fluids and wounds were not different from each other or from urine have been previously reported (Boyd et al., 2008). In our study, a possible sample bias where urine sampling is most common that could be attributed the high MDR isolates in urine samples.

The present study determined high resistance levels (72.2%-98.8%) among *E. coli* isolated from the both countries for the first-line oral antimicrobial agents such as amoxicillin, trimethoprim-sulfamethoxazole, tetracycline, nalidixic acid and amoxicillin-clavulanic acid. Similar rates of resistance have been previously reported in Sudan (Ahmed et al., 2000), Saudi Arabia (Bilal et al., 2001; Al-Tawfiq, 2006), other developing countries (Okeke et al., 2005; Aminizadeh & Kashi, 2011) and developed countries (Sahm et al., 2001; Peralta et al., 2007; Boyd et al., 2008). The high rates of resistance to these commonly used oral antimicrobials makes these agents useless for the empirical treatment of bacterial infection (Bartoloni et al., 2006; Boyd et al., 2008; Namboodiri et al., 2011).

Fluoroquinolones are effective antimicrobial agents used for the treatment of a wide variety of bacterial infections (Drago et al., 2010). High rates of *E. coli* isolates non-susceptible to fluoroquinolones have been reported in many countries as in Thailand (>50%), China (≥70%) and India (>80%) (Hsueh et al., 2010). In the present study, isolates from Sudanese patients revealed relatively high resistance rates (58.4%) to ciprofloxacin, whereas 40% of Saudi Arabia sourced strains were ciprofloxacin resistant. These proportions were higher than previous finding in Saudi Arabia by Al-Tawfig, (2006), who reported ciprofloxacin resistant rates at 33%, among *E. coli* isolates from nosocomial infections. Lee et al. (2010) have determined that 26.6 % of *E. coli* isolates were resistant to ciprofloxacin from Korean patients with acute uncomplicated cystitis. Another report from Ghana estimated that 12 to 18% of fecal *E. coli* isolates are quinolones resistant (Namboodiri et al., 2011). The high rates of resistant in our study have been hypothesized to be related to the inappropriate use of fluoroquinolones (Lee et al., 2010). Also, prolonged use of low dose of fluoroquinolones has been shown to be the most significant risk factor for acquisition of resistance as previously described by Chenia et al. (2006).

In this study, higher resistance rates were observed among *E. coli* strains collected from Sudan than Saudi Arabia for ceftriaxone (64% vs. 31.7%) (p< 0.001) and ceftazidime (35% vs. 20%) (p = 0.004). In a study carried out at two hospitals in Makka city, Saudi Arabia, resistance rates of *E. coli* isolates were recorded as 36.2 % for cefuroxime, 24.6 % for ceftazidime and 18.3% for ceftriaxone (Asghar & Faidah, 2009). Obviously our findings reflected high resistance rate for cefuroxime (92.5%) among Sudan sourced isolates. The high percentage of resistance to cephalosporins notably to cefuroxime in Sudan hospitals is of great concern, since it was found to be much higher than those reported in other parts of the world (Oteo et al., 2002; Peralta et al., 2007). In a13-years hospital based study the rise in cephalosporins resistant *E. coli* was documented due to increased consumption of these drugs (Hsueh et al., 2005).

This study indicated that there were higher significant differences in resistance levels of *E. coli* strains collected from Khartoum state, Sudan comparable to Aseer region, Saudi Arabia in term of gender and age of the patients. Mostly, isolates from different studied population groups in Sudan were more likely to have higher rates of resistance to antimicrobials. Males in Sudan were more resistant than those from Saudi Arabia to cefuroxime (95.5% vs. 39.5%), trimethoprim-sulfamethoxazole (94.4% vs. 74.4%) and gentamicin (38.2% vs. 11.6%) whereas, higher resistance to amoxicillin-clavulanic acid and tobramicin were observed in Saudi Arabia sourced strains (90.7% and 46.5%, respectively) than that from Sudan (48.3% and 21.3%, respectively). These figures are indication of variations in resistance patterns in both countries with relatively higher rates in Sudan. The possible explanation is that this could be due to differences in prescribing patterns, types and quality of antimicrobial agents, as well as availability of drugs. Or perhaps due to better control strategies in the use of antimicrobial drugs, monitoring of resistance levels and updating prescribing policies in Saudi hospitals than in Sudan. Ahmed et al. (2000) have addressed a local problem in the Sudan, where it appears that there is a higher prevalence of antibiotic resistance compared to other countries. Hamdan et al. (2011) proposed that the inappropriate use of antimicrobial in low income countries is perhaps due to the lack of adequate knowledge about drugs and non-availability or non-accessibility of guidelines for therapy. Another study has reported an emerging cause of resistance in Sudan, due to increasing inappropriate and excessive prescribing patterns of antibiotics in health centers (Awad et al., 2006). Furthermore, self medication and poor quality of available antibiotics has been noted in Sudan (Awad & Eltayeb, 2007).

The common MDR phenotypes in our isolates were between amoxicillin/trimethoprim-sulfamethoxazole/cefuroxime/tetracycline/or nalidixic acid/amoxicillin-clavulanic acid. In the early study of antimicrobial resistance in bacterial isolates from patients with diarrhea and urinary tract infection in the Sudan, the most common MDR profile of isolates was reported to ampicillin, amoxicillin, tetracycline, trimethoprim-sulfamethoxazole, sulfonamide, and chloramphenicol (Ahmed et al., 2000). Likewise, these resistant patterns were also noted by others (Bilal et al., 2001; Bartoloni et al., 2006). These findings may not be surprising, since antimicrobial agents are commonly used widely by populations. However, bacteria can develop MDR patterns through different genetic mechanisms (Uma et al., 2009; Ibrahim et al., 2013).

The study concluded that antimicrobial resistance of pathogenic *E. coli* isolates is alarming and emerging in the both regions of Khartoum state, Sudan as well as in Aseer, Saudi Arabia, with different kinds of resistance patterns. Such high rates of resistance among isolates might be due to misuse and unnecessary prescription of antimicrobial drugs in both countries. Continuous monitoring and updating of antimicrobial resistance profile data as well as hospital policy for restriction and prudent use of antimicrobial drugs can reduce the spread of MDR strains. Antimicrobial resistance varied between settings, population and countries therefore, local and international surveillance programs in each region and setting are required.
